# Two adjacent phosphorylation sites in the C-terminus of the channel’s α-subunit have opposing effects on epithelial sodium channel (ENaC) activity

**DOI:** 10.1007/s00424-022-02693-9

**Published:** 2022-05-08

**Authors:** Alexei Diakov, Viatcheslav Nesterov, Anke Dahlmann, Christoph Korbmacher

**Affiliations:** 1 Institut für Zelluläre und Molekulare Physiologie, Friedrich-Alexander-Universität Erlangen-Nürnberg, Waldstr, 6, 91054 Erlangen, Germany; 2 Medizinische Klinik 4 - Nephrologie und Hypertensiologie, Universitätsklinikum Erlangen, Ulmenweg 18, 91054 Erlangen, Germany

**Keywords:** Epithelial sodium channel (ENaC), Serum- and glucocorticoid-induced kinase isoform 1 (SGK1), Dual-specificity tyrosine phosphorylated and regulated kinase 2 (DYRK2), Glycogen synthase kinase 3 beta (GSK3β), *Xenopus laevis* oocytes, Microdissected mouse distal nephron, Patch clamp

## Abstract

How phosphorylation of the epithelial sodium channel (ENaC) contributes to its regulation is incompletely understood. Previously, we demonstrated that in outside-out patches ENaC activation by serum- and glucocorticoid-inducible kinase isoform 1 (SGK1) was abolished by mutating a serine residue in a putative SGK1 consensus motif RXRXX(S/T) in the channel’s α-subunit (S621 in rat). Interestingly, this serine residue is followed by a highly conserved proline residue rather than by a hydrophobic amino acid thought to be required for a functional SGK1 consensus motif according to *in* *vitro* data. This suggests that this serine residue is a potential phosphorylation site for the dual-specificity tyrosine phosphorylated and regulated kinase 2 (DYRK2), a prototypical proline-directed kinase. Its phosphorylation may prime a highly conserved preceding serine residue (S617 in rat) to be phosphorylated by glycogen synthase kinase 3 β (GSK3β). Therefore, we investigated the effect of DYRK2 on ENaC activity in outside-out patches of *Xenopus laevis* oocytes heterologously expressing rat ENaC. DYRK2 included in the pipette solution significantly increased ENaC activity. In contrast, GSK3β had an inhibitory effect. Replacing S621 in αENaC with alanine (S621A) abolished the effects of both kinases. A S617A mutation reduced the inhibitory effect of GKS3β but did not prevent ENaC activation by DYRK2. Our findings suggest that phosphorylation of S621 activates ENaC and primes S617 for subsequent phosphorylation by GSK3β resulting in channel inhibition. In proof-of-concept experiments, we demonstrated that DYRK2 can also stimulate ENaC currents in microdissected mouse distal nephron, whereas GSK3β inhibits the currents.

## Introduction

**Fig. 1:**
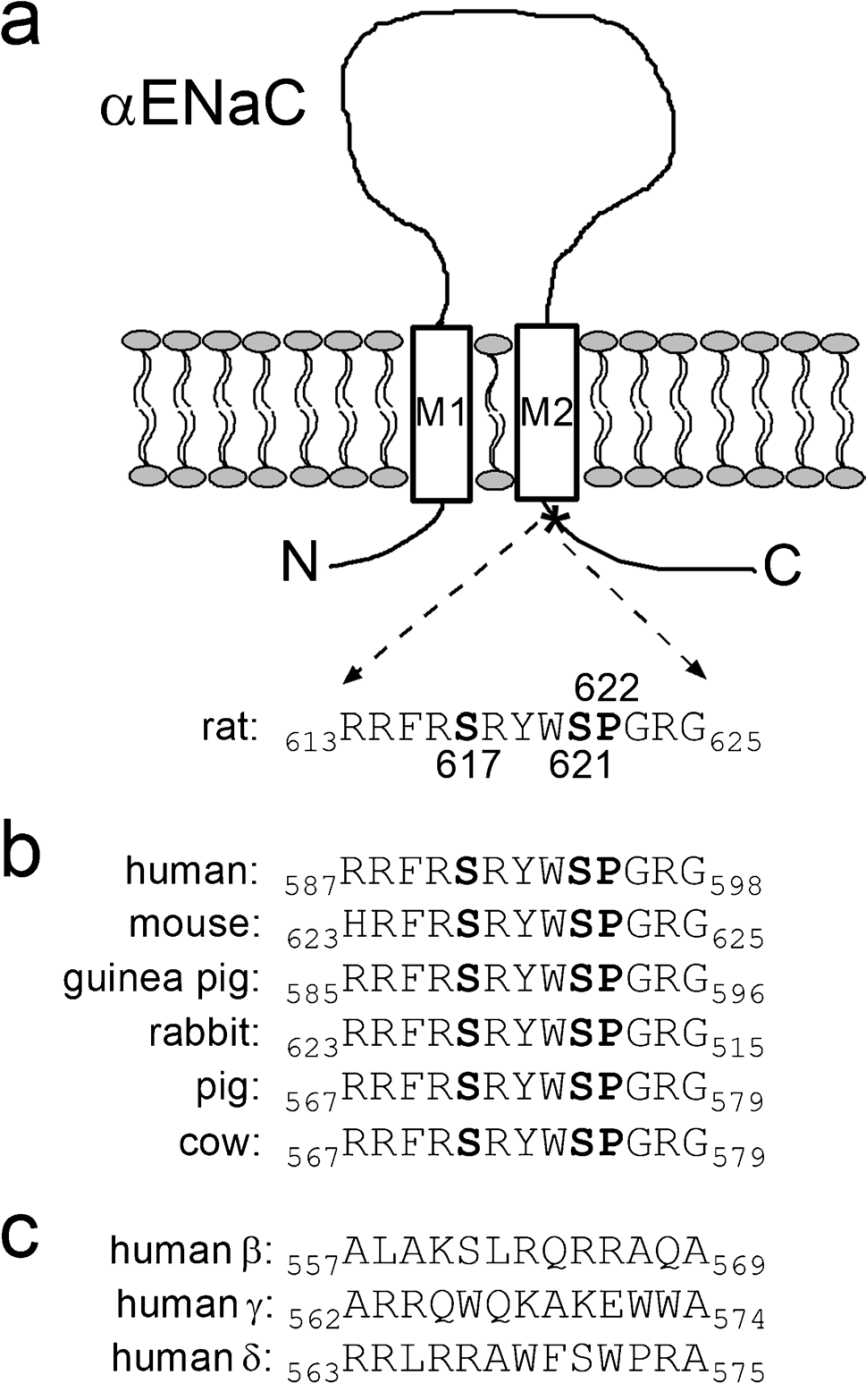
Two serine residues and one proline residue are highly conserved in a C-terminal region of αENaC close to the second transmembrane domain. **a** Schematic representation of αENaC illustrating the extracellular loop, two transmembrane domains (M1 and M2), and intracellular N- and C-termini. The amino acid sequence of rat αENaC (residues 613–625) corresponds to the C-terminal region indicated by a star (*) and contains the serine residues 617 (S617) and 621 (S621) and the proline residue 622 (P622) highlighted in bold. **b** Amino acid sequence alignment of this highly conserved C-terminal region from several mammalian αENaC subunits. The residues homologous to S617, S621, and P622 in rat αENaC are highlighted in bold. **c** Amino acid sequence alignment of homologous C-terminal regions from human β-, γ-, and δENaC subunits

The epithelial sodium channel (ENaC) is the rate-limiting step for Na^+^ absorption in a variety of epithelia and plays a critical role in maintaining Na^+^ balance and controlling long-term blood pressure in mammals [28, 34, 63]. ENaC is probably a heterotrimer consisting of three homologous subunits (α, β, and γ) [60]. Each subunit has two transmembrane domains, a large extracellular loop and cytosolic N- and C-termini (Fig. 1a). In different epithelial tissues, ENaC activity is under the tight control by a range of hormones and local mediators [37, 49, 65].

At the cellular level, ENaC regulation involves a complex interplay of extracellular factors and intracellular signal transduction pathways including a number of protein kinases [3, 37, 65]. Kinases are important regulators of a wide range of cellular processes and consequently may modify ENaC function by acting at many different levels. Effects of kinases on ENaC include the phosphorylation and modification of regulatory proteins associated with ENaC or the phosphorylation of the channel itself with modulatory effects on its interaction with regulatory proteins. A prominent example is the ubiquitin ligase Nedd4-2 [65, 72, 73] which promotes endocytic retrieval and subsequent proteasomal degradation of ENaC. Phosphorylation of Nedd4-2 at specific sites, e.g., by serum- and glucocorticoid-induced kinase 1 (SGK1) [18, 25, 42], reduces its ability to bind to PY-motifs in the cytosolic C-termini of the channel resulting in reduced channel retrieval. This mechanism is thought to contribute to the increase of ENaC expression at the cell surface elicited by aldosterone because the latter has a strong stimulatory effect on SGK1 [12]. On the other hand, phosphorylation of specific sites in the C-termini of β- and γENaC has been reported to modify the channel’s interaction with Nedd4-2 thereby facilitating or impeding Nedd4-2 mediated channel retrieval [22, 41, 68].

In *Xenopus laevis* oocytes, the stimulatory effect of coexpressed SGK1 on ENaC whole-cell currents can be attributed mainly to an increased channel abundance at the cell surface [2, 18, 21, 62]. There is good evidence that this is due to inhibition of Nedd4-2-mediated channel retrieval, but a stimulation of channel forward trafficking may contribute to the effect [2, 45, 46]. This latter concept is also supported by the observation that in the oocyte system, a co-expressed neuronal isoform of SGK increased cell surface expression of homomeric human δENaC known to lack PY-motifs [82]. In addition, we have previously shown that recombinant active SGK1 included in the pipette solution can rapidly activate ENaC in excised outside-out patches from oocytes most probably by increasing the open probability of channels present in the plasma membrane [20]. This direct stimulatory effect on ENaC activity was also observed with recombinant protein kinase B alpha (PKBα) in the pipette solution [21]. Importantly, the stimulatory effect of both kinases was critically dependent on the serine residue S621 in rat αENaC [20, 21]. Originally, the putative phosphorylation site S621 was identified by searching for a conserved SGK/PKB consensus motif RXRXX(S/T) [38, 39] in the cytosolic termini of ENaC. The identified site is located in the carboxyl terminal region of αENaC close to the second transmembrane domain and is highly conserved in mammals (Fig. 1b). A corresponding site is absent in the β-, γ-, and δ-subunits of ENaC (Fig. 1c).

Presently, it remains an open question whether SGK1 and PKBα directly phosphorylate the channel at the identified phosphorylation site S621. Indeed, it is conceivable that the stimulatory effect of the recombinant kinases is mediated by modifying the activity of endogenous kinases or phosphatases present in the patch, thereby indirectly favoring channel phosphorylation at this site. Thus, SGK1 and PKBα may not phosphorylate the channel directly but may mediate their effect by activating or inhibiting an endogenous kinase or phosphatase, respectively. This hypothesis is supported by evidence from *in* *vitro* studies that the RXRXX(S/T) motif has to be followed by a bulky hydrophobic residue to become a preferred SGK/PKB consensus motif [1, 38]. In contrast, in αENaC (Fig. 1b), the RXRXX(S/T) motif is followed by a highly conserved proline residue (P622 in rat). Interestingly, this proline residue makes the preceding serine residue (S621 in rat) a rather poor phosphorylation site for SGK/PKB [1, 85] but a potential phosphorylation site for proline-directed kinases.

The dual-specificity tyrosine-phosphorylation-regulated kinase 2 (DYRK2) is a prototypical proline-directed kinase and a member of a family comprising at least seven mammalian isoforms [5]. DYRK2 has multiple functions and is expressed in a broad range of tissues [16, 75, 84]. These include colonic [81] and renal tubular epithelial cells [10, 78], where DYRK2 may be co-expressed with ENaC. Kinases of the DYRK family (DYRKs) autophosphorylate a critical tyrosine residue in their own activating loop during the translational process at the ribosome [48]. After complete translation and release from the ribosome, tyrosine-kinase-activity is lost and DYRKs phosphorylate their substrates on serine or threonine residues [47]. DYRKs are called proline-directed kinases due to their strong preference for a proline residue at the P + 1 position in combination with an arginine residue at P − 3 position. Thus, the typical phosphorylation recognition sequence of DYRK2 is RXX(S/T)P [8, 16, 70]. This suggests that S621 in the carboxyl terminus of rat αENaC located in the sequence _618_RYWSP_622_ (Fig. 1b) is a good target for DYRK2-mediated phosphorylation.

Interestingly, DYRK2 phosphorylates several substrates which are subsequently recognized and further phosphorylated by the glycogen synthase kinase 3 β (GSK3β) [16]. Among those are eukaryotic initiation factor 2B (eIFB) [83], glycogen synthase [69], collapsin response mediator protein 4 (CRMP4) [15], transcription factors c-Myc, and c-Jun [74]. GSK3β phosphorylation targets are determined by other so-called priming kinases, because GSK3β preferentially phosphorylates its substrates when another phosphoserine (_P_S) or phosphothreonine residue (_P_T) is present four residues C-terminal to the site of GSK3β phosphorylation. Thus, a typical GSK3β recognition motif has the following sequence: SXXX(_P_S/_P_T) [23, 24]. This suggests that phosphorylation of S621 in the α-subunit of rat ENaC may prime the preceding serine residue 617 (S617) for phosphorylation by GSK3β (Fig. 1b).

Therefore, the aim of the present study was to investigate whether DYRK2 and GSK3β can affect ENaC function and whether this involves the two adjacent serine residues S621 and S617 located in the α-subunit of rat ENaC. For this purpose, we tested the effects of recombinant DYRK2 and GSK3β on rat ENaC heterologously expressed in *Xenopus laevis* oocytes using the outside-out patch clamp technique and site-directed mutagenesis. To confirm the oocyte findings in proof-of-concept experiments in native renal tissue, we also studied the effects of recombinant DYRK2 and GSK3β on ENaC currents in patch-clamp recordings from microdissected mouse distal nephron.

## Materials and methods

### cDNA clones

Full-length cDNAs for rat wild-type α-, β-, and ΓENaC [9] and for α_S621A_ENaC [20], α_S617A_ENaC, and α_P622F_ENaC mutants were in pGEM-HE vector. Extension overlap PCR for site directed mutagenesis was used to generate α_S617A_ENaC and α_P622F_ENaC mutants. For the S617A mutation, a mutagenic forward primer with the sequence 5′-CTACGCCGGTTCCGGGCCCGGTACTGGTCTCCA-3′ and a reverse primer with the sequence 5′-TGGAGACCAGTACCGGGCCCGGAACCGGCGTAG-3′ were used to introduce a triplet mutation from CCA at nucleotides 1848–1851 to TTT. To generate α_P622F_ENaC, a mutagenic forward primer with the sequence 5′-GCCGGTACTGGTCTTTTGGACGAGGGGCCAG-3′ and a reverse primer with the sequence 5′-CTGGCCCCTCGTCCAAAAGACCAGTACCGGC-3′ were used to introduce a triplet mutation from GGC at nucleotides 1864–1867 to AGC. Mutations were confirmed by sequence analysis. Linearized plasmids were used as templates for cRNA synthesis using T7 RNA polymerase (mMessage mMachine, Ambion, Austin, TX, USA).

### Isolation of *Xenopus laevis* oocytes and injection of cRNA

Isolation of oocytes was performed essentially as described previously [20, 21, 30, 32, 62]. Oocytes were injected with cRNA using 0.1–0.2 ng of cRNA per ENaC subunit per oocyte. To prevent Na^+^ overloading [33], injected oocytes were incubated in low-sodium modified Barth’s saline (in mM, 1 NaCl, 40 KCl, 60 NMDG-Cl, 0.4 CaCl_2_, 0.3 Ca(NO_3_)_2_, 0.8 MgSO_4_, and 10 HEPES adjusted to pH = 7.4 with HCl) supplemented with 100 U/ml sodium penicillin and 100 µg/ml streptomycin sulfate. Ocytes were studied 48–72 h after injection.

### Recordings in outside-out macropatches excised from *Xenopus laevis* oocytes

Current recordings from outside-out membrane patches were performed essentially as described previously [20, 21, 31, 40, 44] using conventional patch-clamp technique. Patch pipettes were pulled from borosilicate glass capillaries and had a tip diameter of about 5–7 μm after fire polishing. Pipettes were filled with K-gluconate pipette solution (in mM, 90 K-gluconate, 5 NaCl, 2 Mg-ATP, 2 EGTA, and 10 mM HEPES adjusted to pH = 7.28 with Tris). Seals were routinely formed in a low-sodium NMDG-Cl bath solution (in mM, 95 NMDG (N-methyl-D-glucamine)-Cl, 1 NaCl, 4 KCl, 1 MgCl_2_, 1 CaCl_2_, and 10 HEPES adjusted to pH 7.4 with Tris). In this bath solution, the pipette resistance averaged about 3 MΩ. In NaCl bath solution, NMDG-Cl was replaced by 95 mM NaCl. For continuous current recordings, the holding potential was set to − 70 mV using an EPC9 amplifier (HEKA Elektronik, Lambrecht, Germany). Using a 3 M KCl flowing boundary electrode, the liquid junction (LJ) potential occurring at the pipette/NaCl bath junction was measured to be 12 mV (bath positive) [44]. Thus, at a holding potential of − 70 mV, the effective trans-patch potential was − 82 mV. This value is close to the calculated equilibrium potential of Cl^−^ (E_Cl_ =  − 77.4 mV) and K^+^ (E_K_  =  − 79.4 mV) under our experimental conditions. Experiments were performed at room temperature. To change from one bath solution to another, a conventional gravity-fed system controlled by a magnetic valve system (ALA BPS-8) was used in combination with a TIB14 interface (HEKA Elektronik, Lambrecht, Germany). Pulse 8.78 software (HEKA Elektronik, Lambrecht, Germany) was used for data acquisition. Amiloride-sensitive current (∆I_Ami_) in outside-out membrane patches was determined by subtracting the current value recorded in the presence of amiloride (2 μM) from the corresponding value recorded prior to its addition. The current traces were filtered at 200 Hz and sampled at 800 Hz.

### Preparation of mouse renal tubules

For this study, we used 29 male mice (C57BL/6 J originally acquired from Charles River Laboratories, Sulzfeld, Germany) aged 6–8 weeks. Mice were bred and maintained in the animal facility of Friedrich-Alexander-Universität Erlangen Nürnberg (FAU). Mice received a standard diet (Na^+^ content 3.2 g/kg, Cat-No 1310 from Spezialfutter GmbH & Co. KG, Lage, Germany) with free access to tap water. Isolation of renal tubules was essentially performed as described previously [57, 58]. Renal tubules were separated manually using fine forceps. We identified and isolated tubular segments with characteristic branching indicative of the transition from connecting tubules (CNT) to cortical collecting ducts (CCD). Under the dissecting microscope, there are no clear-cut boundaries between CNT and initial CCD. In particular, after transfer of the tubular segments to the perfusion chamber and after opening the tubules, it is difficult to distinguish between CNT and initial CCD. Therefore, we have to assume that our recordings include recordings from CNT as well as from CCD. We did not attempt to distinguish these recordings and pooled the data. The microdissected tubular segments were attached to small pieces of glass coverslips coated with Cell-Tak (Collaborative Research, Bedford, MA, USA) and were transferred to a temperature controlled perfusion chamber (37 °C) mounted on an inverted microscope (Leica DM IRB) to perform patch clamp recordings.

### Whole-cell and outside-out patch clamp recordings from microdissected mouse distal nephron

A computer-controlled EPC-9 patch clamp amplifier (HEKA Elektronik, Lambrecht, Germany) was used to perform conventional whole-cell and outside-out patch clamp recordings as previously described [55–59]. To gain access with the patch pipette to the apical cell membrane, tubules were cut open with a broken glass pipette attached to a micromanipulator. Principal cells expressing ENaC were identified according to their characteristic shape and responsiveness to amiloride. Pipettes were pulled from borosilicate glass capillaries and had a tip diameter of about 1.5 μm after fire polishing. Pipettes were filled with a pipette solution containing the following (in mM): 85 K gluconate, 40 CsOH, 5 Na gluconate, 2 Mg ATP, 2 EGTA Na, 2 MgCl_2_, 20 TEA-OH, and 10 HEPES. Its pH was adjusted to 7.2 with gluconic acid. The bath solution had the following composition (in mM): 145 Na gluconate, 5 K gluconate, 2 CaCl_2_, 5 barium acetate, 1 MgCl_2_, 3 glucose, and 5 HEPES; pH was adjusted to 7.4 with Tris. Pipette resistance measured in the bath solution was about 4–6 MΩ. Seals were formed at the apical surface of principal cells by using gentle suction. Seal resistance ranged from 3 to 10 GΩ. Series resistance was in the order of 10 to 30 MΩ and was not compensated. For continuous whole-cell as well as outside-out current recordings, the holding potential (V_hold_) was set at − 60 mV. In each experiment, ∆I_Ami_ was initially measured in the whole-cell configuration and subsequently in the outside-out configuration provided that membrane patch excision was successful. In both configurations, ∆I_Ami_ was determined by subtracting the current measured in the presence of amiloride (2 μM) from that measured in its absence. The current traces were filtered at 250 Hz and sampled at a rate of 2 kHz. For further analysis, they were digitally re-filtered at 70 Hz. Data were analyzed using the program “Patch for Windows” written by Dr. Bernd Letz (HEKA Elektronik, Lambrecht, Germany).

### Chemicals

Recombinant constitutively active human SGK1 (∆1–60, S422D) and recombinant active full length human DYRK2 were purchased from Biomol GmbH (Hamburg, Germany) as 2 μg (SGK1) and 5 μg (DYRK2) vials in 50 μl stock solution both containing as main components 50 mM Tris–HCl, 0.1 mM EGTA, 0.1% 2-mercaptoethanol, 0.15 mM NaCl, and 270 mM sucrose. SGK1 and DYRK2 pipette solutions were freshly prepared on the day of the experiment by adding stock solutions to 1 ml of the corresponding pipette solutions giving a final SGK1 and DYRK2 concentration of 80 U/ml (for experiments in oocytes) and 20 U/ml (for experiments in microdissected renal tubules). Recombinant GSK3β and a selective GSK3β inhibitor (CHIR99021) were kindly provided by Prof. Philip Cohen (Dundee, UK). GSK3β in a concentration of 1.54 mg/ml was stored in a stock solution containing as main components 50 mM Tris–HCl, 0.1 mM EGTA, 0.1% 2-mercaptoethanol, 0.15 mM NaCl, and 50% glycerol. On the day of the experiment, the GSK3β pipette solution was freshly prepared by adding stock solution to pipette solution giving a final GSK3β concentration of 16 U/ml (for experiments in oocytes) and 8 U/ml (for experiments in microdissected renal tubules). CHIR99021 was stored in DMSO as stock solution with a concentration of 10 mM. On the day of the experiment, stock solution was added to pipette solution giving a final CHIR99021 concentration of 2 μM [54]. To preserve SGK1, DYRK2, and GSK3β activity, the pipette solutions were supplemented with dithiothreitol (Sigma-Aldrich, Taufkirchen, Germany) in a concentration of 0.1 mM in experiments with oocytes and 0.05 mM in experiments with microdissected renal tubules. Control experiments were performed using identical pipette solutions with SGK1, DYRK2, or GSK3β after heat inactivating the solutions at 68° C for 45 min. Moreover, in additional control experiments, we confirmed that dithiothreitol *per se* had no detectable effect on ENaC activity in the concentrations used (data not shown). Amiloride hydrochloride was purchased from Sigma-Aldrich (Taufkirchen, Germany) and was added from an aqueous 10 mM stock solution.

### Statistics

Data are presented as mean values ± SEM; *n* indicates the number of individual recordings. In oocyte experiments, *N* indicates the number of different batches of oocytes used. In animal experiments, *N* is number of mice used in a set of recordings. Data from different oocyte batches and from different animals were pooled. Statistical analysis of nested experiments using *N* and *n* was not performed due to the low number of recordings obtained per animal and oocyte batch. Normal distribution of data was assessed using D’Agostino–Pearson omnibus test. Statistical significance was assessed by an appropriate parametric test: paired or unpaired Student’s *t*-test and Student’s ratio *t*-test. Significance was accepted for *p* < 0.05. Statistical analysis was performed using Graph Pad Prism 5.04.

## Results

### Recombinant DYRK2 stimulates ENaC currents in outside-out patches from *Xenopus laevis* oocytes

**Fig. 2:**
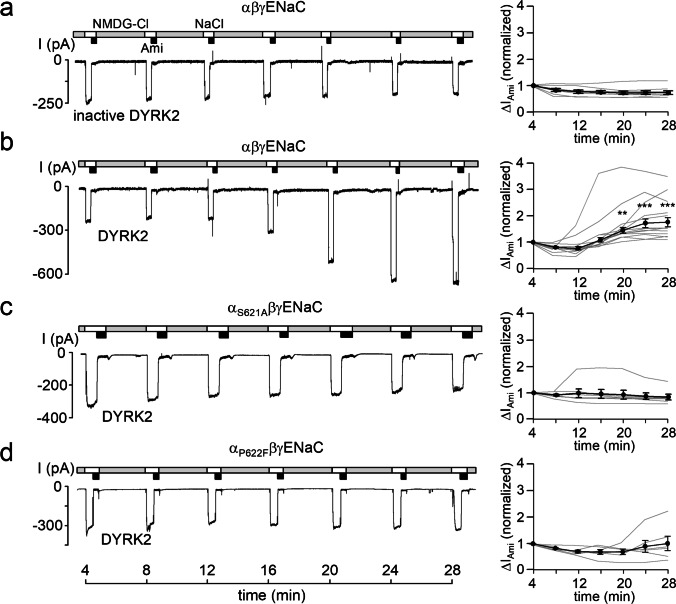
Recombinant DYRK2 stimulates ENaC currents in outside-out patches from *Xenopus laevis* oocytes. **a**, **b**, **c**, and **d**, *Left panels*, representative current traces recorded in outside-out patches of αβγENaC, α_S621A_βγENaC, and α_P622F_βγENaC expressing oocytes at a holding potential (*V*_*hold*_) of − 70 mV. As indicated by the bars, bath solution was changed from a low Na^+^ (NMDG-Cl; [Na^+^] = 1 mM) to a normal Na^+^ containing solution (NaCl; [Na^+^] = 96 mM) without or with amiloride (Ami, 2 μM). Heat-inactivated DYRK2 (inactive DYRK2) or active recombinant DYRK2 (80 U/ml) were included in the pipette solutions as indicated under the traces. *Right panels*, summary of normalized ∆I_Ami_ values obtained from similar experiments as shown in the representative traces (*left panels*). Each grey line corresponds to an individual outside-out patch clamp recording and connects ∆I_Ami_ values obtained at different time points. The black lines in each graph connect average ∆I_Ami_ values (mean ± SEM; αβγENaC/inactive DYRK2, *n* = 8; αβγENaC/DYRK2, *n* = 13; αS_621A_βγENaC/DYRK2, *n* = 7; α_P622F_βγENaC/DYRK2, *n* = 6). ∆I_Ami_ values determined at individual time points were compared with the corresponding initial ∆I_Ami_ value at 4 min using paired Student’s ratio *t*-test. ** *p* < 0.01; *** *p* < 0.001

To investigate whether DYRK2 can modify ENaC activity, we performed patch-clamp recordings using *Xenopus laevis* oocytes expressing α-, β-, and γ-subunits of rat ENaC (αβγENaC). Recordings from outside-out macropatches were started about 4 min after patch excision. To minimize spontaneous channel rundown known to occur in the presence of a high extracellular Na^+^ concentration [80], patches were maintained for most of the time in NMDG-Cl solution containing only 1 mM Na^+^. In this solution, only a negligible inward current was detectable at a holding potential of − 70 mV. In contrast, periodic changes to NaCl bath solution revealed sizeable inward currents consistent with a current component carried by Na^+^ influx via ENaC. This current component was largely inhibited by the application of 2 μM amiloride, a concentration known to specifically inhibit ENaC. Using this protocol, the amiloride-sensitive sodium current (∆I_Ami_) was repeatedly determined to monitor ENaC activity over time. Figure 2a (left panel) shows a representative control recording with heat inactivated DYRK2 (inactive DYRK2) in the pipette solution. Data from similar experiments are summarized in the right panel of Fig. 2a and demonstrate that under control conditions ENaC currents recorded in outside-out patches remain pretty stable for about half an hour. This is consistent with previous control recordings using vehicle control in the pipette solution or other heat-inactivated kinases [20, 21]. In contrast, when catalytically active DYRK2 was included in the pipette solution, ∆I_Ami_ significantly increased by about twofold within ~ 25 min (Fig. 2b). Using a similar experimental approach, we have previously shown that the serine residue S621 in the α-subunit of ENaC is essential for mediating channel activation by SGK1, PKBα, or the phosphatase inhibitor ocadaic acid included in the pipette solution [20, 21]. To test whether this residue is also important for mediating the stimulatory effect of DYRK2, we performed recordings in outside-out patches obtained from oocytes expressing mutant α_S621A_βγENaC in which S621 of the α-subunit was replaced by alanine. No stimulatory effect of DYRK2 was observed on mutant α_S621A_βγENaC (Fig. 2c). Similarly, no stimulatory effect of DYRK2 was detected in outside-out patches obtained from oocytes expressing mutant α_P622F_βγENaC in which P622 of the α-subunit was replaced by phenylalanine (Fig. 2d). These findings indicate that ENaC activation by DYRK2 requires the phosphorylation site S621 and in addition the proline residue P622 most likely as recognition site for the proline-directed kinase which enables it to phosphorylate the preceding serine residue. Phenylalanine was chosen as replacement for P622 to test the functional role of this proline residue and to create an optimal SGK1 consensus site (see next paragraph).

### Recombinant SGK1 fails to stimulate mutant α_P622F_βγENaC

**Fig. 3:**
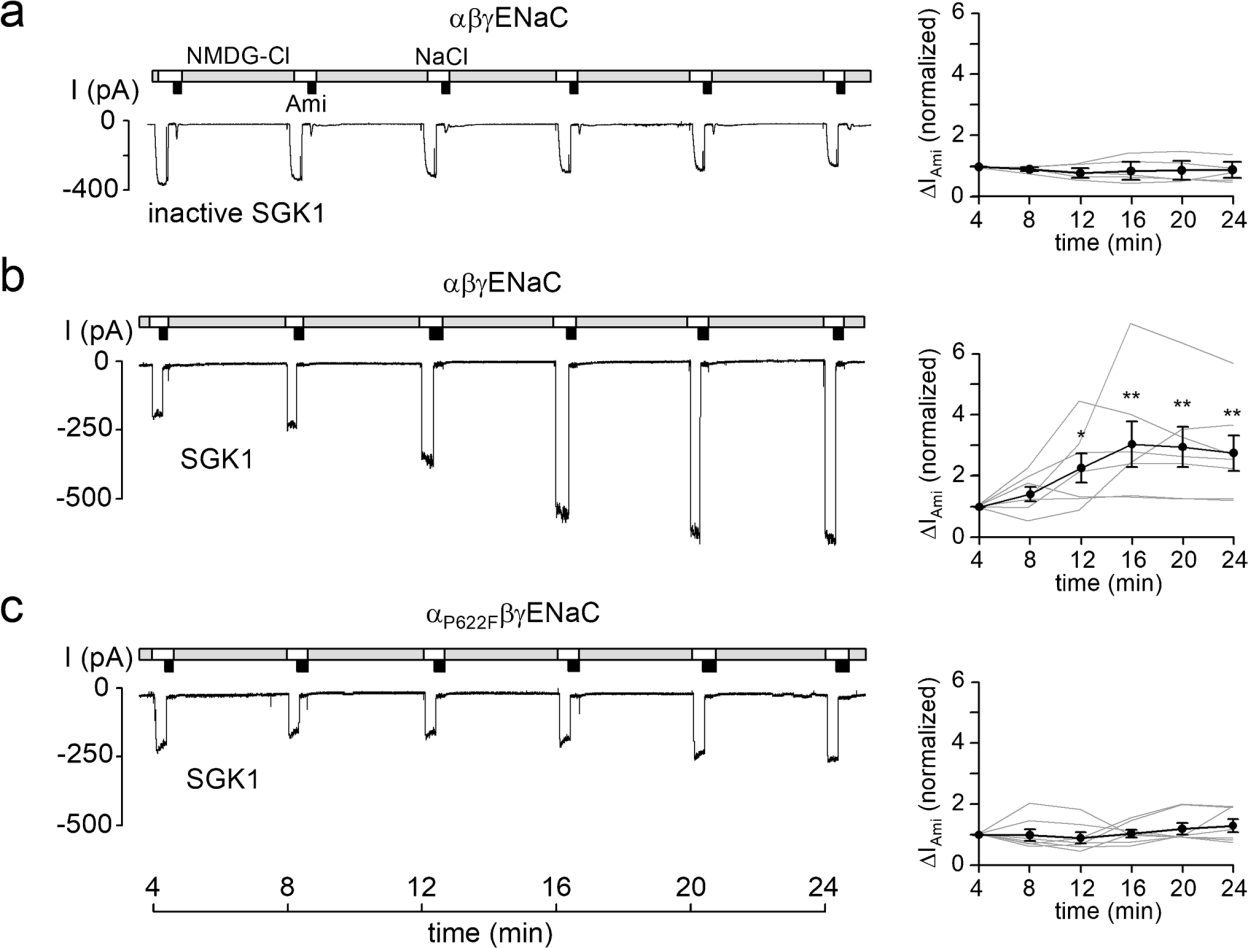
Recombinant SGK1 fails to stimulate α_P622F_βγENaC. *Left panels*, representative current traces recorded in outside-out patches of αβγENaC (**a**, **b**) or α_P622F_βγENaC (*C*) expressing oocytes as described in Fig. 2. Heat-inactivated SGK1 (inactive SGK1) or constitutively active recombinant SGK1 (80 U/ml) were included in the pipette solutions as indicated under the traces. *Right panels*, summary of normalized ∆I_Ami_ values obtained from similar experiments as shown in the representative traces (*left panels*) using the same symbols as in Fig. 2. αβγENaC/inactive SGK1, *n* = 5; αβγENaC/SGK1, *n* = 7; α_P622F_βγENaC/SGK1, *n* = 7. ∆I_Ami_ values determined at individual time points were compared with the corresponding initial ∆I_Ami_ value at 4 min using paired Student’s ratio *t*-test. * *p* < 0.05; ** *p* < 0.01

As stated in the introduction, *in* *vitro* studies indicate that an RXRXX(S/T) motif has to be followed by a large hydrophobic residue to become a functional SGK1 consensus motif [1, 38]. Thus, the P622F mutation in the channel’s α-subunit (α_P622F_ENaC) theoretically converts the original motif (_616_RSRYWS_621_P_622_, Fig. 1) into an optimal SGK1 consensus site (_616_RSRYWS_621_F_622_). Therefore, we asked the question whether SGK1 can activate mutant α_P622F_βγENaC under our experimental conditions. To address this, ENaC activity was assessed by measuring ∆I_Ami_ in outside-out macropatches excised from oocytes expressing αβγENaC or α_P622F_βγENaC. In these recordings, we confirmed our previous findings [20, 21] that ∆I_Ami_ remained stable over time with heat inactivated SGK1 (inactive SGK1) included in the pipette solutions (Fig. 3a) but significantly increased by about threefold within ~ 20 min with catalytically active SGK1 in the pipette solution (Fig. 3b). Importantly, SGK1 failed to stimulate α_P622F_βγENaC (Fig. 3c) despite the predicted optimal SGK1 consensus site of the mutant channel. We cannot exclude the possibility that introducing a hydrophobic residue in the C-terminal domain may cause a structural rearrangement of this flexible loop, thereby preventing SGK1 from accessing its target. However, the well-preserved channel function of α_P622F_ENaC argues against this possibility. Thus, our finding that SGK1 failed to stimulate α_P622F_βγENaC supports the hypothesis that S621 is not directly phosphorylated by SGK1 but that the stimulatory effect of SGK1 is mediated by an indirect effect resulting in the phosphorylation of S621.

### Recombinant GSK3β inhibits ENaC currents

**Fig. 4:**
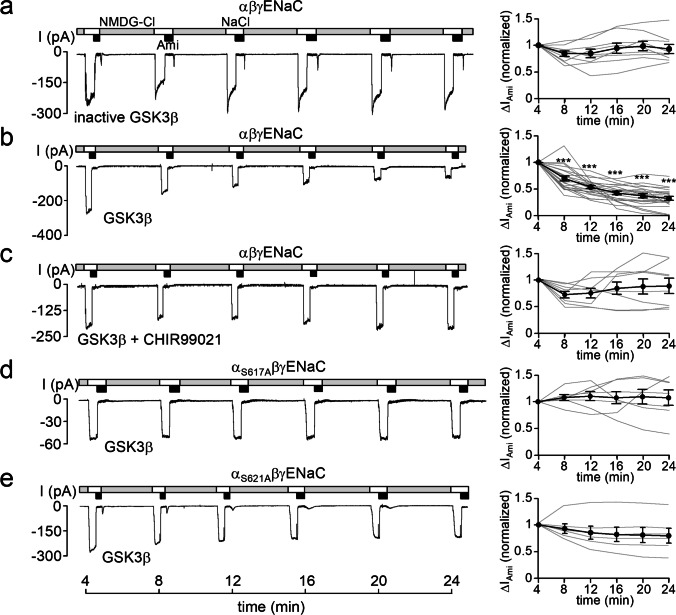
Recombinant GSK3β inhibits ENaC currents. *Left panels*, representative current traces recorded in outside-out patches of αβγENaC (**a**, **b**, **c**), α_S617A_βγENaC (**d**), or α_S621A_βγENaC (**e**) expressing oocytes as described in Fig. 2. Heat-inactivated GSK3β (inactive GSK3β) or active recombinant GSK3β (16 U/ml) were included in the pipette solutions as indicated under the traces. In **c**, the GSK3β inhibitor CHIR99021 (2 µM) was included in the pipette solutions together with GSK3β. *Right panels*, summary of normalized ∆I_Ami_ values obtained from similar experiments as shown in the representative traces (*left panels*) using the same symbols as in Fig. 2. αβγENaC/inactive GSK3β, *n* = 9; αβγENaC/GSK3β, *n* = 20; αβγENaC/GSK3β + CHIR99021, *n* = 8; αS_617A_βγENaC/GSK3β, *n* = 7; αS_621A_βγENaC/GSK3β, *n* = 6. ∆I_Ami_ values determined at individual time points were compared with the corresponding initial ∆I_Ami_ value at 4 min using paired Student’s ratio *t*-test. *** *p* < 0.001

As noted in the introduction, phosphorylation of S621 may prime the preceding S617 for phosphorylation by GSK3β, because the latter preferentially phosphorylates its substrates four residues N-terminal to a phosphoserine (_P_S) or phosphothreonine residue (_P_T) [23, 24]. Therefore, we tested the effect of GSK3β on ENaC activity using the same approach as described above for DYRK2 and SGK1. In control experiments, we detected no effect of heat-inactivated GSK3β (inactive GSK3β) on ENaC currents (Fig. 4a). However, catalytically active GSK3β significantly reduced ∆I_Ami_ to less than 50% of its original value (Fig. 4b). In contrast, the inhibitory effect of GSK3β was not observed, when the specific GSK3β inhibitor CHIR99021 [54] was included in the pipette solution in addition to GSK3β. This supports the conclusion that the inhibitory effect GSK3β on ENaC is specific and due to its kinase activity. Importantly, GSK3β failed to inhibit ENaC currents in outside-out patches from oocytes expressing α_S617A_βγENaC (Fig. 4d) or α_S621A_βγENaC (Fig. 4e). Thus, the inhibitory effect of GSK3β depended on the presence of S617 and also on the presence of S621. This suggests that phosphorylation of S621 is required as a priming site for GSK3β to phosphorylate S617.

### Recombinant DYRK2 stimulates α_S617A_βγENaC

**Fig. 5:**
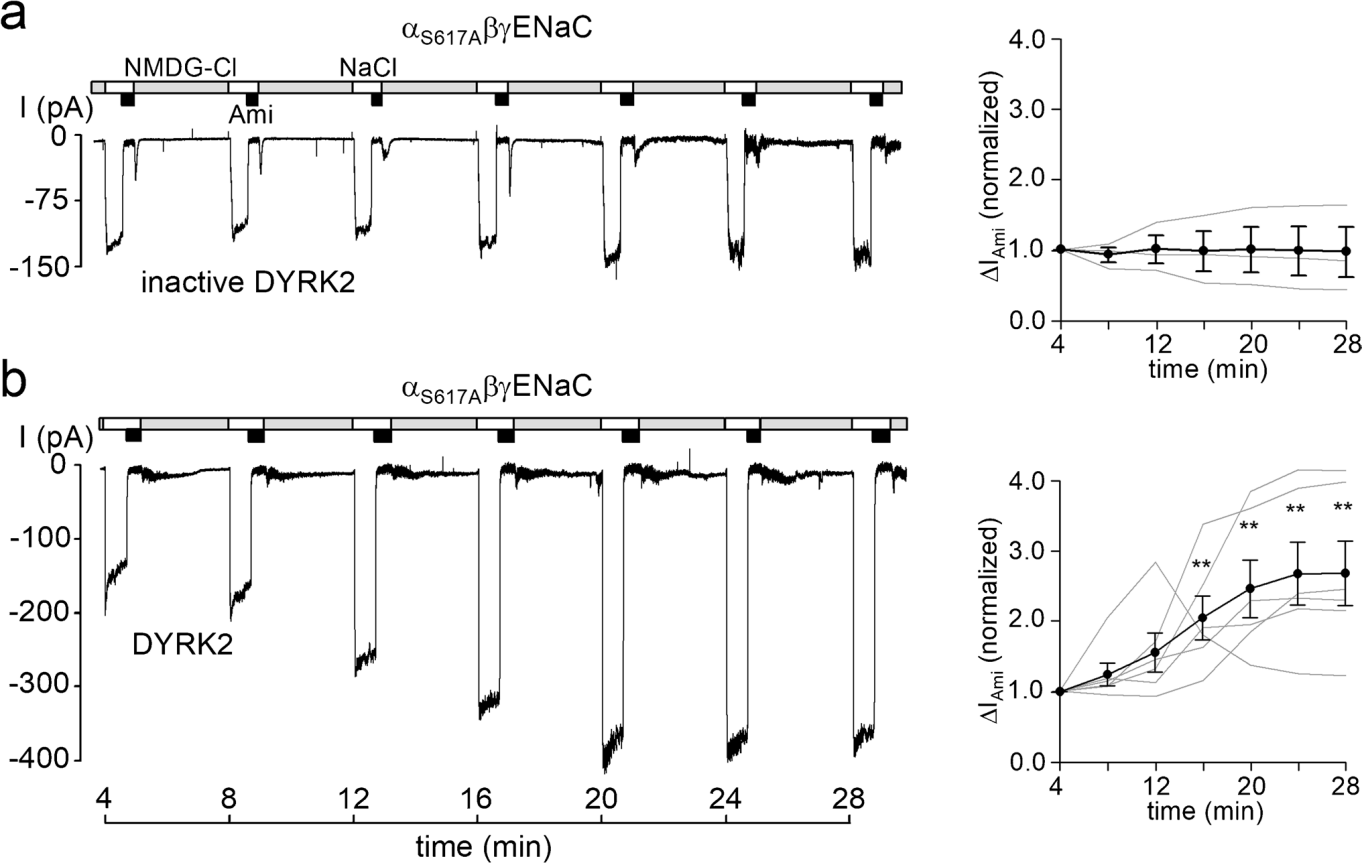
Recombinant DYRK2 activates α_S617A_βγENaC. **a** and **b**, *left panels*, representative current traces recorded in outside-out patches of α_S617A_βγENaC expressing oocytes as described in Fig. 2. Heat-inactivated DYRK2 (inactive DYRK2) or active recombinant DYRK2 (80 U/ml) were included in the pipette solutions as indicated under the traces. *Right panels*, summary of normalized ∆I_Ami_ values obtained from similar experiments as shown in the representative traces (*left panels*) using the same symbols as in Fig. 2. αS617AβγENaC/inactive DYRK2, *n* = 3; α_S617A_βγENaC/DYRK2, *n* = 6. ∆I_Ami_ values determined at individual time points were compared with the corresponding initial ∆I_Ami_ value at 4 min using paired Student’s ratio *t*-test. ** *p* < 0.01

Since mutating S617 or S621 in the channel’s α-subunit abolished the inhibitory effect of GSK3β, we wondered whether the stimulatory effect of DYRK2 was preserved in outside-out patches from oocytes expressing α_S617A_βγENaC. In control recordings, we demonstrated that inactive DYRK2 had no significant stimulatory effect on α_S617A_βγENaC (Fig. 5a). Importantly, the stimulatory effect of catalytically active DYRK2 on α_S617A_βγENaC was fully preserved with a ~ 2.5-fold increase of ∆I_Ami_ within ~ 25 min (Fig. 5b). Taken together with the findings shown in Fig. 2, this indicates that the stimulatory effect of DYRK2 is dependent on S621 and P622, but independent of S617.

### DYRK2 can stimulate ENaC in outside-out patches from principal cells of microdissected mouse distal nephron

Our oocyte data indicate that DYRK2 and GSK3β can stimulate and inhibit ENaC activity, respectively, and that these effects are mediated by specific phosphorylation sites in the C-terminus of the channel’s α-subunit. To explore a possible regulatory role of these kinases in native renal tissue, we tested the effects of DYRK2 and GSK3β on ENaC currents in microdissected mouse distal nephron using an established experimental technique [55–59]. Tubular epithelial cells were approached from the apical membrane, and amiloride-sensitive ENaC currents (ΔI_Ami_) were recorded in the whole-cell and outside-out configuration of the patch-clamp technique. Microdissected and split open tubular fragments of the connecting tubule (CNT) and cortical collecting duct (CCD) were used which are known to express ENaC in principal cells. For the purpose of this study, we did not distinguish between recordings from CNT and CCD and pooled the data from all successful experiments (see Experimental Procedures).

**Fig. 6:**
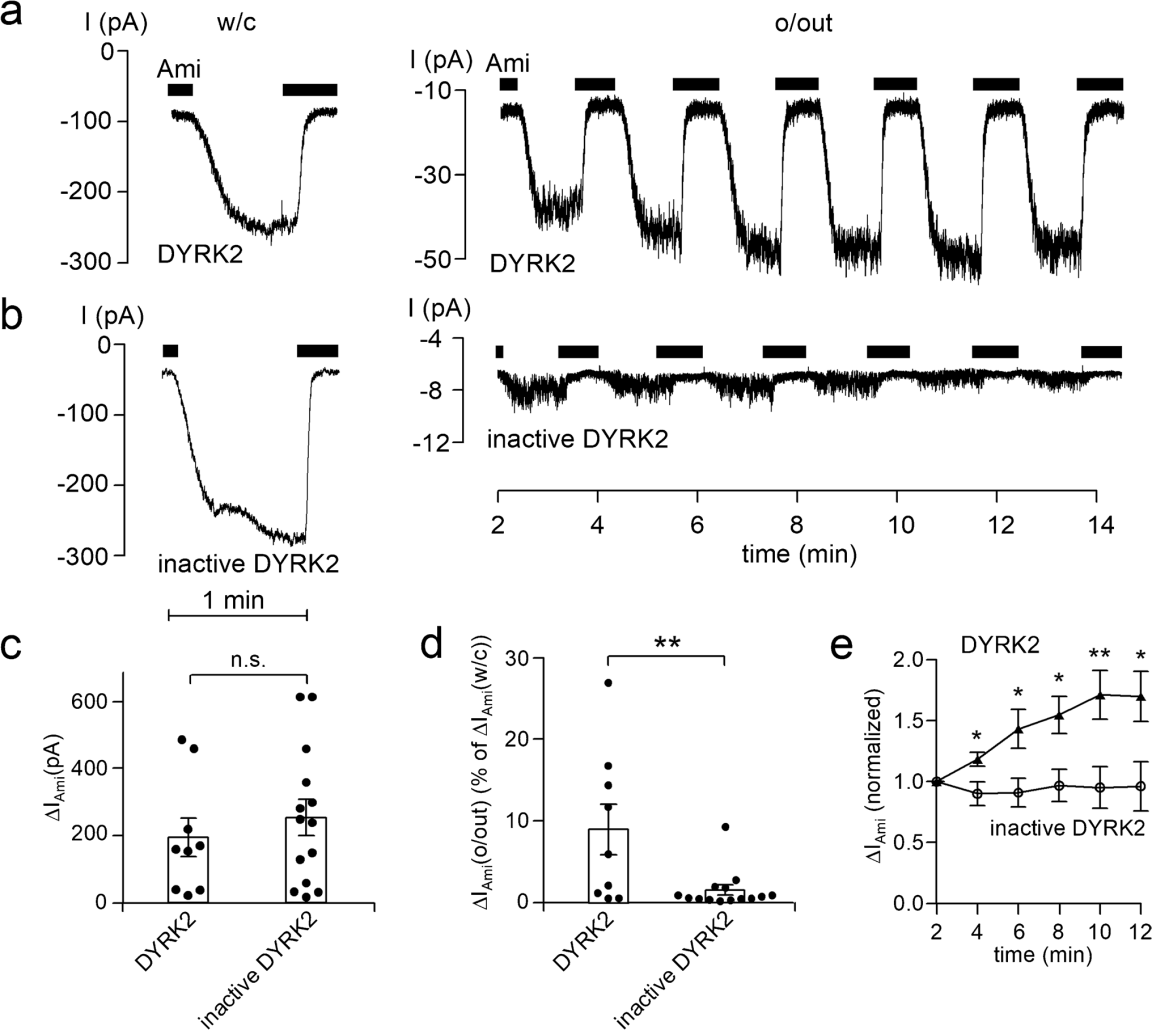
DYRK2 stimulates ENaC currents in outside-out patches excised from the apical membrane of principal cells in split open microdissected mouse renal tubules. **a** and **b** Representative current traces from whole-cell (*left panels*) and subsequent outside-out (*right pan*els) patch-clamp recordings at a continuous *V*_*hold*_ of − 60 mV. Black bars indicate the presence of amiloride (Ami, 2 μM) in the bath solution. Active recombinant DYRK2 (40 U/ml; **a**) or heat-inactivated DYRK2 (inactive DYRK2; **b**) were included in the pipette solutions as indicated under the traces. **c–e** Summary of data from similar experiments as shown in the representative traces (**a** and **b**, *left panels*) with DYRK2 (*n* = 9) or inactive DYRK2 (*n* = 14) in the pipette solution. In **c** and **d**, black dots correspond to measurements from individual patches, and open columns with error bars represent mean values ± SEM. ∆I_Ami_ values shown in **c** were determined in the whole-cell configuration. Data shown in **d** represent ∆I_Ami_ values from outside-out patches (∆I_Ami_(o/out)) expressed as percentage of the corresponding whole-cell ∆I_Ami_ values (% of ∆I_Ami_ (w/c)). DYRK2, *n* = 9; inactive DYRK2, *n* = 14. In **c** and **d**, statistical significance was assessed by unpaired Student’s *t*-test and unpaired Student’s ratio *t*-test, respectively. **e** Averaged normalized ΔI_Ami_ recorded in outside-out patches as illustrated in **a** (*right panel*) at different times after patch excision with DYRK2 (solid triangles, *n* = 9) or inactive DYRK2 (open circles, *n* = 14) in the pipette solution. In **e**, ∆I_Ami_ values determined at individual time points were compared with the corresponding initial ∆I_Ami_ value at 2 min using paired Student’s ratio *t*-test. * *p* < 0.05; ** *p* < 0.01; n.s. not significant

At the beginning of the experiments, whole-cell currents were recorded at a holding potential of − 60 mV (Fig. 6a and 6b, left panels). Recordings were started in the presence of amiloride (2 μM). Washout of amiloride revealed an ENaC-mediated inward current component which was rapidly inhibited upon reapplication of amiloride. A detectable amiloride-sensitive whole-cell current was taken as evidence that the cell under investigation was a principal cell expressing ENaC. After determining ∆I_Ami_ in the whole-cell configuration, we routinely tried to excise an outside-out patch and to record from the same cell ∆I_Ami_ also in the outside-out configuration. In successful attempts, we monitored ∆I_Ami_ over time by repeated application and washout of amiloride (Fig. 6a and 6b, right panels). Representative current traces from experiments with active recombinant DYRK2 or heat-inactivated DYRK2 in the pipette solution are shown in Fig. 6a and 6b, respectively. Data from similar recordings are summarized in Fig. 6c, d, and e. Interestingly, the amiloride-sensitive whole-cell currents were not significantly different in the two groups with ∆I_Ami_ averaging 253 ± 54 pA (*n* = 14; *N* = 11) in control experiments and 193 ± 57 pA (*n* = 9; *N* = 8) in experiments with active DYRK2 included in the pipette solution (Fig. 6c). A possible explanation for this is that active DYRK2 included in the pipette solution does not reach ENaC in the plasma membrane in a sufficient concentration. Interestingly, recordings performed in the outside-out configuration revealed a significant difference between the two groups. In the control group with inactive DYRK2, the initial ∆I_Ami_ measured in the outside-out configuration averaged 1.54 ± 0.63% (*n* = 14) of the corresponding ∆I_Ami_ determined in the whole-cell mode (Fig. 6d). This percentage roughly reflects the ratio of the area of the pipette tip to the area of the entire apical cell membrane. Importantly, in experiments with active DYRK2 in the pipette solution, the initial ΔI_Ami_ in outside-out patches was increased to 8.96 ± 3.08% of ΔI_Ami_ determined in the corresponding whole-cell recordings. This value was significantly higher than that observed in control recordings with heat-inactivated DYRK2 (*p* < 0.01) (Fig. 6d). Moreover, in outside-out patches with active DYRK2 in the pipette solution ΔI_Ami_ further increased over time (Fig. 6a, right panel) reaching on average ~ 160% of its initial value within ~ 10 min (Fig. 6e). In contrast, in control recordings in outside-out patches with inactive DYRK2 in the pipette solution, ΔI_Ami_ remained relatively stable (Fig. 6b, right panel; Fig. 6e). These findings indicate that DYRK2 can stimulate ENaC currents in outside-out patches from native renal tubules. Apparently, the main stimulatory effect occurs within the initial 2–3 min needed to establish the outside-out configuration with some further stimulation after patch excision.

### GSK3 has an inhibitory effect on ENaC in outside-out patches from principal cells of microdissected mouse distal nephron.

**Fig. 7:**
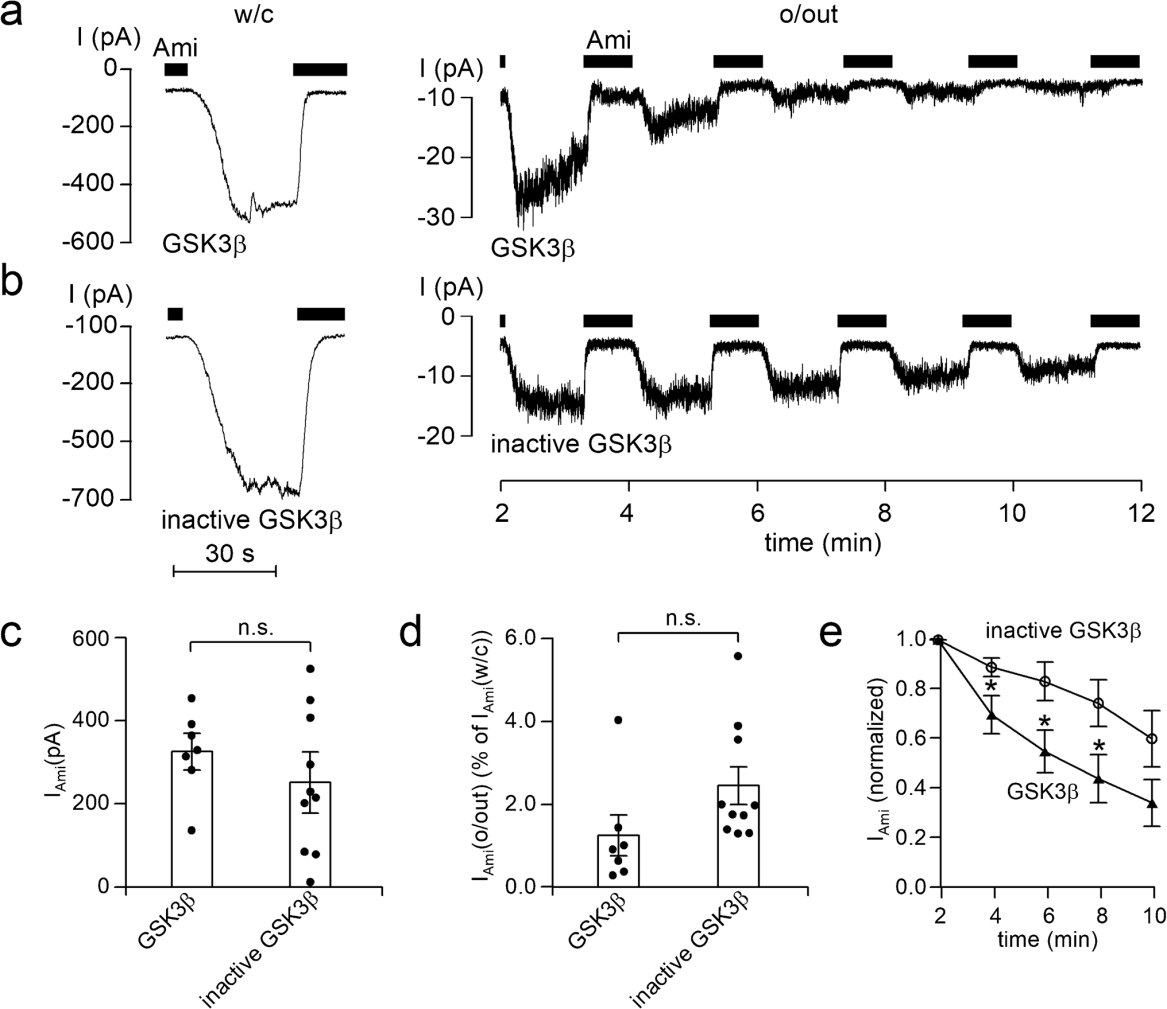
GSK3β inhibits ENaC currents in outside-out patches excised from the apical membrane of principal cells in split open microdissected mouse renal tubules. **a** and **b** Representative current traces from whole-cell (*left panels*) and subsequent outside-out (*right panels*) patch-clamp recordings from experiments similar to those shown in Fig. 6. Active recombinant GSK3β (8 U/ml; **a**) or heat-inactivated GSK3β (inactive GSK3β; **b**) were included in the pipette solutions as indicated under the traces. **c–e** Summary of data from similar experiments as shown in the representative traces (**a** and **b**, *left panels*) with GSK3β (*n* = 7) or inactive GSK3β (*n* = 10) in the pipette solution. In **c** and **d**, black dots correspond to measurements from individual patches, and open columns with error bars represent mean values ± SEM. ∆I_Ami_ values shown in **c** were determined in the whole-cell configuration. Data shown in **d** represent ∆I_Ami_ values from outside-out patches (∆I_Ami_(o/out)) expressed as percentage of the corresponding whole-cell ∆I_Ami_ values (% of ∆I_Ami_ (w/c)). GSK3β, *n* = 7; inactive GSK3β, *n* = 10. In **c** and **d**, statistical significance was assessed by unpaired Student’s *t*-test and unpaired Student’s ratio *t*-test, respectively. **e** Averaged normalized ΔI_Ami_ recorded in outside-out patches as illustrated in **a** (*right panel*) at different times after patch excision with GSK3β (solid triangles, *n* = 7) or inactive GSK3β (open circles, *n* = 10) in the pipette solution. In **e**, ∆I_Ami_ values determined at individual time points were compared with the corresponding initial ∆I_Ami_ value at 2 min using paired Student’s ratio *t*-test. * *p* < 0.05; n.s. not significant

In similar experiments, we also tested the effect of GSK3β on ENaC in microdissected mouse distal nephron. Constitutively, active recombinant GSK3β or heat-inactivated GSK3β were added to the pipette solution. Representative traces from these experiments are depicted in Fig. 7a and 7b, respectively, and the results are summarized in Fig. 7c, d, and e. Similar to the experiments with DYRK2, ΔI_Ami_ measured in the whole-cell configuration was not different in the group with active GSK3β compared to ΔI_Ami_ in the control group with inactive GSK3β (Fig. 7a and 7b, left traces; Fig. 7c). The initial ΔI_Ami_ determined in the outside-out configuration appeared to be slightly reduced in the group with active GSK3β. The ratio between the initial ΔI_Ami_ in outside-out patches and the corresponding ΔI_Ami_ in the whole-cell configuration averaged 1.24 ± 0.49% (*n* = 7; *N* = 6) in experiments with GSK3β in the pipette solution and 2.43 ± 0.45% (*n* = 10; *N* = 7) in control experiments with inactive GSK3β (Fig. 7d). This difference did not reach statistical significance (*p* = 0.096). However, ΔI_Ami_ declined more rapidly in outside-out patches with active GSK3β in the pipette solution than in patches with inactive GSK3β (Fig. 7a and 7b, right panels). Within 4 min after patch excision, relative ΔI_Ami_ was significantly lower in outside-out patches with active GSK3β in the pipette solution than relative ΔI_Ami_ in outside-out patches from the control group with inactive GSK3β (Fig. 7e). After 8 min, this difference was even more pronounced, and ΔI_Ami_ had declined to 46 ± 1.1% (*n* = 7; *N* = 6) of its initial value in the experiments with active GSK3β but only to 78 ± 0.9% (*n* = 10; *N* = 7) in control experiments with inactive GSK3β (*p* = 0.02). These findings indicate that GSK3β can inhibit ENaC in native renal tubules.

## Discussion

The key findings of the present study are the following: (i) DYRK2 stimulated and GSK3β inhibited ENaC activity in outside-out patches from oocytes; (ii) the stimulatory effect of DYRK2 depended on the amino-acid residues S621 and P622 in the C-terminus of rat αENaC which supports the hypothesis that S621 is a phosphorylation site for a proline directed kinase; (iii) the inhibitory effect of GSK3β depended on both S617 and S621, consistent with the idea that phosphorylation of S621 primes S617 to be phosphorylated by GSK3β; (iv) the S617 phosphorylation site is necessary for channel inhibition by GKS3β but not required for channel activation by DYRK2; (v) in proof-of-concept experiments we demonstrated a stimulatory effect of DYRK2 and an inhibitory effect of GSK3β on ENaC activity also in microdissected mouse distal nephron.

This study confirms our previous finding that in outside-out patch clamp recordings from oocytes an intact S621 phosphorylation site in the C-terminus of rat αENaC is critical for mediating acute channel activation by SGK1 included in the pipette solution [20]. So far, it remained unclear whether SGK1 directly phosphorylates S621 or whether a downstream kinase is needed to mediate the S621-dependent stimulatory effect of SGK1. The findings of the present study support the hypothesis that due to a proline residue at the P + 1 position, the serine residue S621 is a phosphorylation site for proline-directed kinases, e.g., DYRK2. Indeed, replacing P622 by phenylalanine prevented ENaC activation by DYRK2, highlighting the functional importance of this proline residue to make S621 a suitable phosphorylation site for this prototypical proline-directed kinase. Interestingly, the P622F mutation also inhibited the stimulatory effect of SGK1, despite converting the original motif into an optimal SGK1 consensus site (_616_RSRYWS_621_F_622_). Moreover, the proline residue following S621 makes the latter an unlikely SGK1 phosphorylation site according to *in* *vitro* studies [1, 38, 85]. Taken together, this argues against a direct phosphorylation of S621 by SGK1, although its stimulatory effect is lost when S621 is replaced by an alanine [20, 21].

We have previously shown that the stimulatory effect of SGK1 can be mimicked by including the broad range phosphatase inhibitor okadaic acid or protein phosphatase inhibitor type 2 (PIP2) in the pipette solution [20]. Importantly, the stimulatory effect of phosphatase inhibition on ENaC also critically depended on S621 and was abolished by mutating this site [21]. Moreover, ENaC activity in outside-out patches decreased to very low levels, when Mg^2+^ was omitted from the pipette solution. This is consistent with the concept that an endogenous Mg^2+^-dependent kinase activity is present in the patch and is involved in maintaining baseline ENaC activity [20]. Inclusion of phosphatase inhibitors probably shifts the balance between endogenous kinase and phosphatase activity in the patch to favor phosphorylation of the stimulatory S621 site. Similarly, inclusion of SGK1 or DYRK2 in the pipette solution is likely to favor phosphorylation of S621 resulting in ENaC activation above baseline level. In case of DYRK2, this is most likely a direct effect which can be inhibited by mutating the proline residue P622. The latter residue is critical for DYRK2 to recognize the S621 phosphorylation site. In contrast, SGK1 may indirectly lead to S621 phosphorylation by stimulating an endogenous oocyte kinase or by inhibiting an endogenous phosphatase. In both cases, the resulting ENaC activation is due to increased phosphorylation of S621 as indicated by the finding that mutating this residue abolished the stimulatory effect of both SGK1 and DYRK2. The identity of the endogenous oocyte kinase and phosphatase involved in the phosphorylation and dephosphorylation of S621, respectively, is presently unknown.

The concept of an indirect effect of SGK1 on S621 is also consistent with our previous observation that plasma membrane cholesterol removal abolishes the acute stimulatory effect of SGK1 on ENaC in outside-out patches [40]. Cholesterol removal is likely to compromise the function of cholesterol-rich lipid raft microdomains, which are thought to serve as signaling platforms for ENaC and may be important for the channel’s interaction with associated regulatory proteins. Thus, the finding that cholesterol depletion prevents ENaC stimulation by SGK1 suggests that additional regulatory proteins, e.g., an endogenous kinase or phosphatase, are required to mediate the stimulatory effect of SGK1 on the channel.

Our findings in microdissected tubules provide proof of principle that the prototypical proline-directed kinase DYRK2 can activate ENaC not only in the oocyte expression system but also in native renal tissue. DYRK2 is known to be expressed in the kidney [16] including in renal tubular epithelial cells co-expressing ENaC [10, 78]. Moreover, using RNA-seq analysis, we recently confirmed expression of DYRK2 in cultured mouse cortical collecting duct (CCD) cells from the mCCD_cl1_ cell line (unpublished observation). This cell line is known to express ENaC and is an established CCD model to study ENaC-mediated transepithelial sodium transport [26, 50–52]. Whether DYRK2 is the physiologically relevant kinase or whether other proline-directed kinases are involved in ENaC activation by targeting S621 remains to be investigated. Regulating mechanisms and stimuli able to modify the expression and activity of DYRK2 are still incompletely understood [16]. Thus, it is presently unclear how DYRK2 may be regulated to modify ENaC function according to physiological needs. Moreover, it is conceivable that in the presence of a suitable baseline kinase activity, the degree of S621 phosphorylation is determined by adjusting the activity of a phosphatase dephosphorylating this site rather than by modifying the kinase activity.

At present, it is unclear how phosphorylation at S621 mechanistically causes channel activation. Our previous findings indicate that in outside-out patches, the acute stimulatory effect of SGK1 on ENaC is not due to the insertion of additional channels into the plasma membrane. Instead, constitutively active SGK1 in the pipette solution seems to activate a population of near-silent channels present in excised outside-out patches from ENaC expressing oocytes [20]. Thus, phosphorylation at S621 probably affects ENaC gating and turns previously silent channels into channels with a rather high open probability. Interestingly, this is reminiscent of proteolytic ENaC activation observed in single-channel recordings from outside-out patches exposed to trypsin or chymotrypsin in the bath solution [19, 29]. Proteolytic channel activation is a unique feature of ENaC [64, 65], but the underlying mechanisms and the identity of the physiologically relevant proteases remain incompletely understood. There is good evidence that proteases stimulate ENaC by cleaving specific sites in the extracellular loops of its α- and γ-subunits [37]. This cleavage causes the release of inhibitory tracts which probably leads to a conformational change resulting in channel activation [35]. Recently published cryo-EM structural data of ENaC indicate that specific binding sites are present to allow a close interaction of the α- and γ-inhibitory tracts with their respective subunits [60, 61]. However, it is still unclear how the occupancy of these binding sites alters channel conformation. No cryo-EM structural information is presently available regarding the C-termini of ENaC including the S621 residue in α-ENaC. Thus, at present, it remains purely speculative to postulate that a phosphorylation at this site may result in a conformational change similar to that induced by proteolytic channel activation. It will be an interesting task for future studies to explore a possible link between proteases and kinases in acutely regulating ENaC activity at the level of the plasma membrane.

As stated in the introduction, DYRK2 is known to act as priming kinase for GSK3β [16]. Our findings support the hypothesis that phosphorylation of S621 primes S617 for phosphorylation by GSK3β. Interestingly, GSK3β inhibited ENaC expressed in *X. laevis* oocytes and in microdissected tubules. Moreover, in the oocyte expression system, its effect depended on both S617 and on S621. In contrast, the stimulatory effect of DYRK2 was fully preserved when S617 was mutated and S621 remained intact. Thus, phosphorylation of S617 by GSK3β may serve as feedback mechanism to limit ENaC activation induced by phosphorylation of S621. Our functional data provide indirect evidence that phosphorylation of these residues is relevant for ENaC regulation. It remains a challenge for future studies to demonstrate direct phosphorylation of these sites and to elucidate how the degree of phosphorylation varies according to different physiological conditions.

GSK3β was first discovered to phosphorylate glycogen synthase, a final enzyme in the glycogen synthesis pathway. GSK3β is expressed in many tissues [13, 14] including ENaC expressing renal epithelial cells [10, 11, 78]. Indeed, we recently confirmed co-expression of ENaC and GSK3β in mCCD_cl1_ cells using RNA-seq analysis (unpublished observation). Phosphorylation of glycogen synthase by GSK3β leads to its inactivation [13]. Inhibition of GSK3β mediates the effect of insulin on glycogen synthesis [17]. Protein kinase B alpha (PKBα, named also Act1), induced by insulin, phosphorylates GSK3β at serine residue S9, thereby inactivating it [66, 67]. Interestingly, insulin has been reported to activate ENaC [4, 6, 7, 53, 71, 77]. The stimulatory effect of insulin on ENaC may be mediated partially by PKBα, because it was shown that this kinase increases ENaC abundance in the plasma membrane [43] and acutely activates ENaC in outside-out patches [21]. The latter effect depends on S621. Since we have shown that ENaC is inhibited by GSK3β, it is tempting to speculate that inhibition of GSK3β by PKBα contributes to ENaC activation by insulin. Moreover, activation of ENaC by other kinases known to inactivate GSK3β may be partially attributed to their inhibitory effects on this kinase. For instance, SGK1 [66] and the protein kinase A (PKA) [76] can inactivate GSK3β through phosphorylation at S9. Thus, SGK1 and PKA, induced by aldosterone [27, 34] and vasopressin [36, 79], respectively, may activate ENaC at least in part by inactivation of GSK3β. Taken together, GSK3β may be involved in ENaC regulation as a tonic inhibitory factor and/or may serve as an inhibitory feedback mechanism after ENaC activation by phosphorylation at S621.

In summary, our study indicates that phosphorylation of S621 by a proline-directed kinase, e.g., DYRK2, stimulates ENaC activity and primes S617 to be phosphorylated subsequently by GSK3β which limits the stimulatory effect of the initial phosphorylation and results in channel inhibition. Moreover, our findings provide proof of principle that DYRK2 and GSK3ß can activate and inhibit ENaC also in microdissected renal tubules, respectively. This supports the concept that the opposing effects of the two adjacent phosphorylation sites in the C-terminus of the channel’s α-subunit play a role in acute regulation of ENaC activity in native tissue.

## Data Availability

The datasets generated during and/or analyzed during the current study are available from the corresponding author on reasonable request.
