# Correction to: Two adjacent phosphorylation sites in the C-terminus of the channel’s α-subunit have opposing effects on epithelial sodium channel (ENaC) activity

**DOI:** 10.1007/s00424-022-02717-4

**Published:** 2022-06-09

**Authors:** Alexei Diakov, Viatcheslav Nesterov, Anke Dahlmann, Christoph Korbmacher

**Affiliations:** 1 Institut für Zelluläre und Molekulare Physiologie, Friedrich-Alexander-Universität Erlangen-Nürnberg, Waldstr, 6, 91054 Erlangen, Germany; 2 Medizinische Klinik 4 - Nephrologie und Hypertensiologie, Universitätsklinikum Erlangen, Ulmenweg 18, 91054 Erlangen, Germany

**Correction to: Pflügers Archiv - European Journal of Physiology (2022)**

10.1007/s00424-022-02693-9

Originally, the article was published with an error. The affiliation of the author Christoph Korbmacher should be "Institut für Zelluläre und Molekulare Physiologie, Friedrich-Alexander-Universität Erlangen-Nürnberg, Waldstr, 6, 91054 Erlangen, Germany”.

The original article has been corrected.

